# Do Not Give Up on Ossified Tuberculum Sellae Meningioma – Vision Restored

**DOI:** 10.7759/cureus.11258

**Published:** 2020-10-30

**Authors:** Carlos Candanedo, Moshe Attia

**Affiliations:** 1 Neurosurgery, Hadassah-Hebrew University Medical Center, Jerusalem, ISR; 2 Neurosurgery, Sheba Medical Center, Tel Aviv, ISR

**Keywords:** tuberculum sella meningioma, blindness, retractorless brain surgery, optic nerve

## Abstract

This case report accompanies a video of the treatment of a Tuberculum sellae meningioma (TSM) in a woman that presented with left eye near-blindness. The senior author conducted the operation via a pterional retractorless approach, and the patient had a full recovery. This case report discusses the unique anatomy of conchal sphenoid sinus, ossified TSM with very calcified consistency, and retractorless brain microsurgery. The resection of ossified TSM is still safe and viable with adequate microsurgical techniques and skull base instruments without compromising the neurovascular structure and with good neurological and visual outcomes for the patient.

## Introduction

Tuberculum sellae meningioma (TSM) often compresses the optic nerves and the optic chiasm, and patients with this condition present with visual deterioration. Surgical treatment consists of gross total resection and decompression of the optic apparatus to achieve visual improvement. There is a correlation between the preoperative visual function and the prognosis-if the patient is unable to detect hand movement, count fingers, or detect light; the prognosis is inferior [1]. This case report presents a video of ossified TSM in a 57-year-old woman who presented with left eye near-blindness. The senior author conducted the operation via a pterional retractorless approach, and the patient fully recovered.

## Case presentation

Video 1 presents a review of the case and documents the operation.

**Video 1:** Two-dimensional operative video of ossified tuberculum sella meningioma Timestamps and topics: 0:00 - Introduction, 00:07- Clinical Presentation, 00:18 - Neurological exam, 00:44 - Neuro-imaging findings, 1:03 - Rationale of the procedure, 1:18 - Risk of the procedure and its potential benefits, 1:33- Alternatives and why they were not chosen, 1:59 - Positioning, 2:30 - Any necessary equipment, 2:37 - Key surgical steps, 3:03 - Dural opening, 3:18 - Tumor exposure, 5:29 - Closure, 5:39 - Disease Background, 5:47 - Clinical outcome, 5:52 - Imaging outcome, 6:14 – Conclusion

Video transcript

0:00 Introduction

This video demonstrates an ossified TSM resection through a pterional approach.

00:07 Clinical Presentation

A 57-year-old woman, otherwise healthy, presented to the emergency department due to sustained left eye blindness and headache lasting three days.

00:18 Neurological Exam

On physical examination, the patient was fully conscious, cooperative, and oriented. She had left visual acuity of 1/60 and a relative afferent pupillary defect, suggesting a severe optic neuropathy, confirmed by a neuro-ophthalmologic evaluation. There was no other cranial nerve deficit or focal motor deficit.

00:44 Neuro-Imaging Findings

A computed tomography scan of her head showed an ossified suprasellar lesion (2.4 cm), and a characteristic conchal-type sphenoid sinus. Magnetic resonance imaging (MRI) of her brain revealed the suspected TSM severely compressing the left optic nerve.

1:03 Rationale of the Procedure

Given the patient’s characteristic conchal-type sphenoid sinus, an endonasal endoscopic approach was not preferred. We decided to perform a left extended pterional craniotomy for a trans-Sylvian-sub frontal approach for a gross total resection.

1:18 Risk of the Procedure and its Potential Benefits

The benefit of the procedure is tumour removal to attempt visual improvement. The risks of the procedure include vascular injury, optic nerve injury, frontal lobe contusion, seizures, pituitary gland injury, and infection.

1:33 Alternatives and Why They Were Not Chosen

An alternative approach would be via an endoscopic endonasal route, but due to the patient’s conchal-type sphenoid sinus, this approach is not preferred. We could also opt to observe rather than operate, especially given the ossified meningioma. However, given the patient’s severe visual deterioration, we decided to give the patient a chance to recover her vision by removing the tumour.

1:59 Positioning

The surgery was performed with the patient in a supine position, with her head rotated 30 degrees, slightly extended to reflect the frontal lobe away from the anterior skull base, making a retractorless corridor for the approach. The surgeon made a standard curvilinear skin incision over the hairline. A pterional craniotomy was performed with one unique burr hole over the temporal squama and extended over the orbital rim for a sub frontal corridor to add to the trans-Sylvian corridor.

2:30 Any Necessary Equipment

The procedure used skull base instruments, microdissectors, and the ShearTip™ of the Cavitron Ultrasonic Surgical Aspirator (CUSA®; Integra LifeSciences, Plainsboro, NJ).

2:37 Key Surgical Steps

The key surgical steps included a left pterional craniotomy with frontal extension for adequate exposure of the pathology. The Sylvian fissure was dissected to achieve cerebrospinal fluid (CSF) drainage and brain relaxation. The surgeon then performed devascularization, detachment, and debulking of the tumour. Next, the surgeon dissected the tumour from the optic nerve, optic chiasm, internal carotid artery, and the brain.

3:03 Dural Opening

After extradural drilling of the sphenoid wing, the dura was opened in a C-shaped fashion, with exposure and dissection of the Sylvian fissure to achieve brain relaxation until the optic nerve was identified, which was severely compressed by the tumour.

3:18 Tumor Exposure

We identified the left internal carotid artery and the oculomotor nerve. The tumour was not invading the optic canals, so there was no need for optic canal unroofing. The surgeon performed bipolar coagulation of the insertion of the tumour from the tuberculum sellae to devascularize and detach the tumour and perform central debulking with the ultrasonic aspirator. We used the ShearTip of the CUSA to remove this ossified tumour. Next, the contralateral optic nerve was exposed, and we dissected the tumour from its border. The right internal carotid artery was also identified in its inferolateral aspect. From the carotid-optic triangle, the pituitary stalk was also observed as compressed by the tumour. With a ball tip dissector, the surgeon carefully dissected between the tumour and the left optic nerve. The surgeon also performed an arachnoid dissection between the tumour and the optic nerve. Once we identified the plane between these two structures, the tumour was carefully separated from the optic nerve without injuring the pituitary stalk and elevating the inferior part of the tumour. Next, the surgeon performed coagulation of the tumour’s last insertion from the tuberculum sellae. Then, we identified the left ophthalmic artery entering the optic canal below the optic nerve. The surgeon dissected the posterior part of the tumour that had adhered to the optic chiasm, and in doing so, disconnected and completed the tumour removal. The anterior cerebral arteries remained intact, as well as the right and left optic nerves and optic chiasm.

5:29 Closure

Once hemostasis was verified, primary closure of the dura was performed using Monocryl® Suture 4-0, and the site was sealed TachoSil® and DuraSeal®.

5:39 Disease Background

Disease background is not presented in the video.

5:47 Clinical Outcome

The patient was discharged home. On postoperative neuro-ophthalmologic follow-up a few months later, we noted she had experienced complete recovery of her vision with no other neurological deficits.

5:52 Imaging Outcome

**Figure 1:**
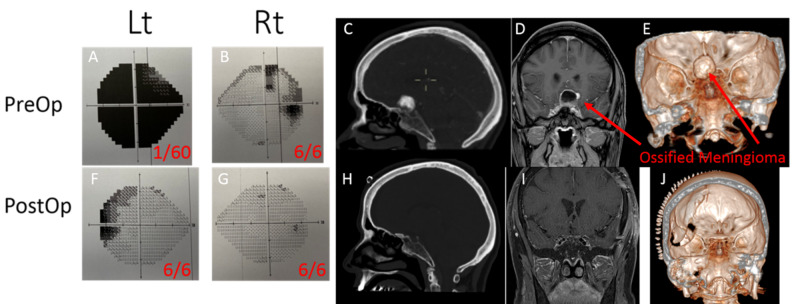
Pre and post-operative Humphrey visual field tests, acuity tests and brain imaging Figure 1: (A, B) Preoperative visual field and acuity tests. (C, D, E) Preoperative brain CT and MRI. (F, G) Postoperative visual field and acuity tests. (H, I, J) Post-operative brain CT and MRI. Abbreviations: CT, computed tomography; MRI, magnetic resonance imaging.

Her nine-month postoperative MRI showed complete removal of the ossified TSM with no signs of frontal encephalomalacia due to the retractorless pterional approach. Her pathology report was consistent with a Psammomatous meningioma, World Health Organization Grade I (Figure 1).

6:14 Video Conclusion

Surgical resection of the ossified TSM was attainable with adequate microsurgical techniques and the use of skull base instruments such as the CUSA ShearTip, without compromising any neurovascular structures and with good neurological and visual outcomes for the patient.

## Discussion

To treat this patient, we did not use the endoscopic endonasal approach because the patient had a conchal sphenoid sinus [2], making the approach especially challenging [3] as well as hindering our ability to identify the internal carotid arteries and the optic nerves. We, therefore, preferred to use a pterional retractorless approach [4] using the trans-Sylvian and sub frontal routes with no violation of the brain parenchyma and maintained excellent control of the neurovascular structures.

We demonstrate our resection technique for this unique meningioma with its hard, very calcified consistency that compressed the patient’s left optic nerve, causing near-blindness. While the unique anatomy of a conchal sphenoid sinus is not suitable for an endonasal endoscopic approach, the presence of an ossified TSM with very calcified consistency is not a hopeless scenario-physicians should not give up. Our case reinforces the notion that there is always the chance to improve vision, especially if the operation occurs soon after visual deterioration, but hope remains even if the meningioma has ossified.

## Conclusions

This case report presents the treatment of ossified tuberculum sellae meningioma in a woman that presented with left eye near-blindness. She was operated upon via pterional retractorless aproach and had a full recovery. Clinicians must remember that the optic nerve may have a great potential for recovery and even with the poor visual condition, we should always give a chance and provide decompression of the optic nerve. Moreover, even if the meningioma looks very calcified on imaging studies, it still may be defragmented with a special sheared CUSA tip which should be present in the armament of the neurosurgeon
